# Lymphatic invasion of micropapillary cancer cells is associated with a poor prognosis of pathological stage IA lung adenocarcinomas

**DOI:** 10.3892/ol.2014.2284

**Published:** 2014-06-25

**Authors:** HIROSHI HIRANO, HAJIME MAEDA, YUKIYASU TAKEUCHI, YOSHIYUKI SUSAKI, RYOZI KOBAYASHI, AKIO HAYASHI, NAOKO OSE, TOSHIHIKO YAMAGUCHI, SOICHIRO YOKOTA, MASAHIDE MORI

**Affiliations:** 1 Department of Pathology, Toneyama National Hospital, Toyonaka, Osaka 560-8552, Japan; 2 Department of Surgery, Toneyama National Hospital, Toyonaka, Osaka 560-8552, Japan; 3 Department of Internal Medicine, Toneyama National Hospital, Toyonaka, Osaka 560-8552, Japan

**Keywords:** lung adenocarcinoma, micropapillary, immunohistochemistry, pathological stage IA

## Abstract

The cancer cells of lung adenocarcinoma with a micropapillary pattern (MPP) have been found to frequently invade lymphatic vessels, and the prognosis of patients with lung adenocarcinoma with an MPP is poor. In the present study, the cancer cells of lung adenocarcinomas containing an MPP were found to express vimentin more extensively than those in lung adenocarcinoma without an MPP. The contribution of cancer cells in the MPP component to adenocarcinoma lymphatic invasion was assessed using vimentin as a marker. Vimentin expression was analyzed in the cancer cells present in each lymphatic vessel and compared with the expression of vimentin in the cancer cells in the adenocarcinomas without an MPP component. The results showed that the cancer cells in the lymphatic vessels expressed vimentin more extensively than those in the adenocarcinoma components without an MPP, suggesting that cancer cells derived from an MPP component are present in the lymphatic vessels. By contrast, the area of the MPP component in each adenocarcinoma was <25%. These findings suggest that cancer cells in MPP components have a high capacity to invade lymphatic vessels and that their high invasive capacity may be associated with a poor prognosis in patients with adenocarcinoma with an MPP component.

## Introduction

Lung cancer is the leading cause of cancer-related mortality in the USA and worldwide (1). In the USA, the age-adjusted incidence and mortality rates of lung cancer (between 2004 and 2008) is 62 and 51.6 per 100,000 men and women per year, respectively, with these rates higher in men than in women (2). Of all types of lung cancer, adenocarcinoma is the most common histological subtype, accounting for ~40% of all lung cancers, and is increasing in frequency (1). Although surgery may be possible, a certain population of patients with adenocarcinomas show poor prognosis. A micropapillary pattern (MPP) in lung adenocarcinoma has been reported to be an indicator of a poor prognosis in lung adenocarcinoma (2–4). This poor prognosis has been shown to be associated with a higher frequency of lymphatic and venous invasion in lung adenocarinomas with an MPP (2–4). However, whether the cancer cells in the MPP have a role in the invasion of cancer cells into lymphatic and venous vessels has yet to be elucidated. Therefore, the present study aimed to investigate the role of cancer cells in the MPP in lymphatic and venous invasion in lung adenocarinoma using pathological stage IA lung adenocarcinoma samples.

## Patients and methods

### Patients

The present study included 218 patients (102 males and 116 females) with pathological stage IA lung adenocarcinoma who underwent complete resection of their tumors at Toneyama National Hospital (Toyonaka, Japan) between January 2002 and December 2010. None of the patients received neoadjuvant chemotherapy or radiotherapy. All patients underwent dissection of the bifurcation and the ipsilateral mediastinal lymph nodes, and pathological examination revealed no metastasis in these locations. Furthermore, computed tomography and magnetic resonance images showed no metastasis in any of the patients. Pathological stage was determined according to the tumor-node-metastasis classification of the International Union Against Cancer (7th edition; tumor size <3.0 cm, T1N0M0 and no pleural invasion) (5). Clinical information for each patient was obtained through reviewing medical charts. The follow-up periods ranged between 12 and 113 months (mean, 55 months). Informed consent was obtained from each patient. The present study was approved by the ethics committee of Toneyama National Hospital (approval number: 1305).

### Histology

**Figure 1:**
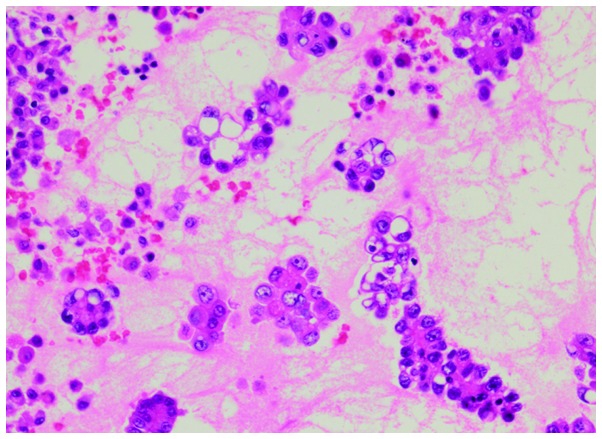
Micropapillary pattern in lung adenocarcinoma exhibiting papillary tufts lacking a central fibrovascular core (hematoxylin and eosin stain; magnification, ×20)

The diameters of the resected tumors were measured and the longest diameter was regarded as the tumor diameter. The tumors were fixed in 0.01 M phosphate-buffered saline containing 10% formalin (pH 7.4) and several paraffin-embedded tumor blocks were generated from each tumor. Tumor sections (4-mm thick) were then generated from each tumor block. Sections were either used for hematoxylin and eosin staining or immunohistochemistry. Tumors were histologically classified according to the International Association for the Study of Lung Cancer (6). MPPs were identified as small tufts without a fibrovascular core, present in the alveolar spaces or in spaces encased within thin walls of connective tissues (Fig. 1), as described previously (2–4).

### Immunohistochemistry

Immunohistochemical staining was performed using an avidin-streptavidin immunoperoxidase method with anti-human E-cadherin (dilution, 1:50; Novocastra, Newcastle, UK), anti-human vimentin (dilution, 1:200; Dako, Glostrup, Denmark) and anti-human D2-40 (dilution, 1:20; Dako) mouse monoclonal antibodies. Antigen retrieval was performed through incubating the deparaffinized sections in cell condition 1 solution (Ventana Medical Systems, Inc., Tucson, AZ, USA) at a low degree (the slide temperature is controlled at 36°C). Immunohistochemical staining was then performed using an automated Benchmark system (Ventana Medical Systems) according to the manufacturer’s instructions.

The grades of immunohistochemical staining for E-cadherin and vimentin were determined according to the proportion (p) of positive cells as follows: 0, p<5% positive cells; 1+, 5≤p<30% positive cells; 2+, 30≤p<70% positive cells; 3+, p≥70% positive cells.

In order to analyze the expression of vimentin in the cancer cells which had invaded into lymphatic vessels, two serial tumor sections were established and the sections were either stained for vimentin or D2-40. When cancer cells were found in a D2-40-positive lymphatic vessel in a section stained for D2-40, the grade of vimentin-positive staining of these cancer cells was analyzed in the other section stained for vimentin as described above.

### Statistical analyses

Survival curves were plotted using the Kaplan-Meier method and the survival rates of the two groups were analyzed using the log-rank test. Data for more than three samples are presented as the mean ± standard error of the mean. Student’s t-test was used to compare data between two groups. The differences in frequencies between two groups were analyzed using the t^2^ test. All statistical analyses were performed using the Excel Statistics 2012 software package (SSRI, Tokyo, Japan) for Windows. P<0.05 was considered to indicate a statistically significant difference.

## Results

### Survival rates and clinicopathological chracteristics of patients with adenocarcinomas

In the present study, tumors were classified into three groups based on the proportion of the MPP area in the adenocarcinoma according to a study by Miyoshi *et al* (3). In brief, the classification was performed as follows: 0%, adenocarcinoma with no MPP component; <5%, adenocarcinoma with a focal MPP component; and >5%, adenocarcinoma with an apparent MPP component. The numbers of patients with adenocarcinoma containing no MPP component, a focal MPP component and an apparent MPP component were 171 (78.4%), 29 (13.3%) and 18 (8.3%), respectively.

**Figure 2:**
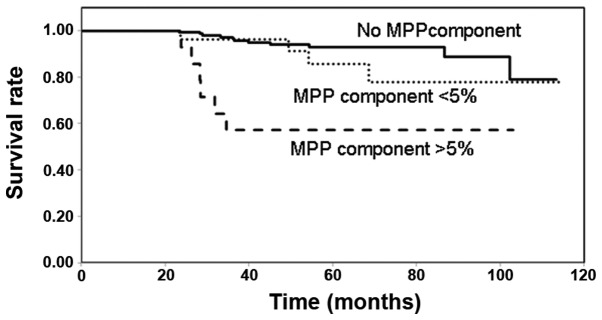
Survival rates of patients with adenocarcinoma containing no MPP component, those containing a focal MPP component (MPP area, <5%) and those with an apparent MPP component (MPP area, >5%). MPP, micropapillary pattern.

Fig. 2 shows the survival rates of the patients with adenocarcinoma in the different MPP groups. The survival rate of the patients with adenocarcinoma containing an apparent MPP component was significantly decreased compared with that in the patients with no MPP component or those with a focal MPP component. The survival rates of the patients with adenocarcinoma containing no MPP component and those with a focal MPP component were not significantly different.

The clinicopathological characteristics of the patients with a low survival rate (the apparent MPP component group) and those with a higher survival rate (the non- and focal MPP component groups) were compared (Table I). The patients in the apparent MPP component group were predominantly male and had a papillary predominant histology, histologically moderate differentiation and a higher frequency of cancer cell lymphatic invasion.

### Expression of E-cadherin and vimentin

**Figure 3:**
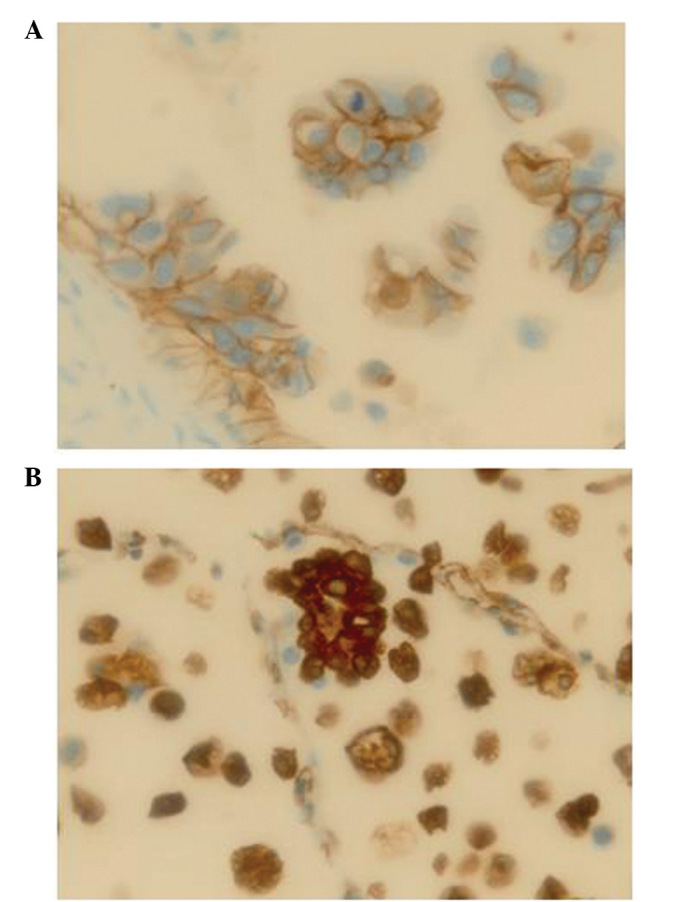
Immunohistochemical (A) E-cadherin and (B) vimentin staining of a micropapillary pattern component in lung adenocarcinoma (magnification, ×40).

Epithelial-mesenchymal transition (EMT) has an important role in cancer metastasis (7). During EMT, cancer cells lose E-cadherin-mediated cell-cell adhesion and acquire characteristics of mesenchymal cells, including the expression of vimentin. In the present study, the tumors with an apparent MPP component showed a higher frequency of lymphatic invasion (Table I). Therefore, the present study analyzed the expression of E-cadherin and vimentin in the MPP components and the components without MPP in the patients in the apparent MPP component group, as well as those in the no MPP component group (Table II, Fig. 3). In the patients in the apparent MPP component group, the cancer cells in the MPP components exhibited E-cadherin expression similar to that in components without MPP. However, the cancer cells in the MPP components expressed vimentin more extensively than those in the components without MPP.

**Figure 4:**
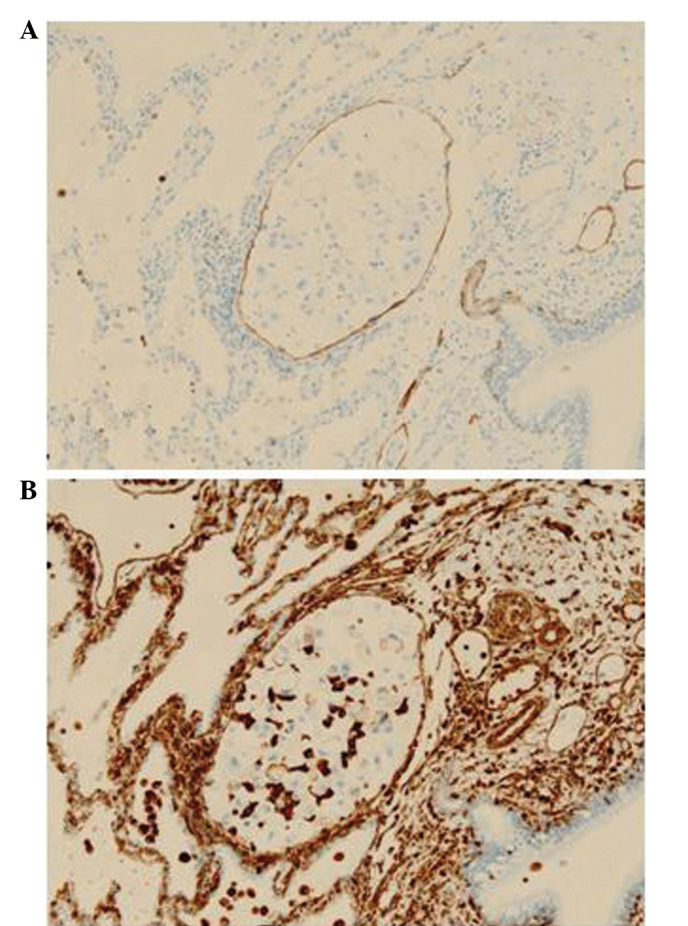
Vimentin expression in the cancer cells in a lymphatic vessel. Serial sections were stained for either (A) D2-40 to identify a lymphatic vessel or (B) vimentin (magnification, ×20).

In the patients in the apparent MPP component group, cancer cell lymphatic invasion was identified in 5/18 cases. In order to assess which component of the tumor contributed to the cancer cell lymphatic vessel invasion, the level of vimentin expression was analyzed in the MPP and non-MPP components, as well as in the cancer cells in each lymphatic vessel (Table III; Fig. 4). The proportion of the MPP component in the five adenocarcinomas with lymphatic invasion was <25 and these MPP components exhibited vimentin expression at grade 3+. In three of the adenocarcinomas with no MPP components, vimentin expression was found to be negative, and in two others, vimentin expression was observed to be grade 2+ and 1+. The grade of vimentin expression in each lymphatic vessel was higher than that in the non-MPP component, indicating that the cancer cells detected in the MPP component are also present in the lymphatic vessels.

## Discussion

In the present study, the survival of patients with adenocarcinoma containing an apparent MPP component was found to be worse than that in the patients with adenocarcinoma containing no MPP component or a focal MPP component. Furthermore, adenocarcinoma with an apparent MPP component had a higher frequency of cancer cell lymphatic invasion. These results confirm the findings reported previously (2,3). The poor prognosis of patients with adenocarcinoma with an apparent MPP component may be associated with the higher frequency of lymphatic invasion of the cancer cells in this type of adenocarcinoma.

Kamiya *et al* (4) reported that cancer cells in the MPP component express E-cadherin and exhibit cell-cell adhesion. This is in accordance with the findings of the present study. In addition, to the best of our knowledge, the present study has provided the first evidence that cancer cells in the MPP component express vimentin more extensively than those in the non-MPP component in adenocarcinoma. Vimentin is a marker of mesenchymal cells (8); therefore, this finding suggests that the cancer cells in the MPP component may transform into mesenchymal cells.

The present study also investigated whether cancer cells in lymphatic vessels were derived from the MPP component or the non-MPP component. The results suggested that all of the lymphatic vessels containing cancer cells had cancer cells derived from the MPP component. In each adenocarcinoma exhibiting cancer cell lymphatic invasion, the MPP component occupied <25% of the tumor area. Therefore, it is likely that the cancer cells in the MPP component have a greater invasive potential compared with those in the non-MPP component.

In conclusion, the present study identified that adenocarcinoma with an MPP component had histological predominance of the papillary dominant type and the moderately differentiated type. These findings are consistent with those of previous studies (2,3).

## Figures and Tables

**Table I:** Clinicopathological characteristics of the patients with adenocarcinoma with an apparent MPP component compared with those with no MPP component or a focal MPP component.

Clinicopathological characteristic	Apparent MPP component (n=18) (MPP component; >5%)	No or focal MPP component (n=200) (MPP component: 0 or <5%)
Age	68.1±2.4	64.1±9.5
Gender		
Male	13 (72.2)	89 (44.5)
Female	5 (27.8)	111 (55.5)
Size (cm)	1.8±0.1	1.8±0.1
Histological type	MPP-positive	MPP-negative
AIS; mixed mucinous/nonmucinous	0 (0)	2 (1)
AIS; mucinous	0 (0)	2 (1)
AIS; nonmucinous	0 (0)	36 (18)
MIA; nonmucinous	0 (0)	20 (10)
Papillary predominant	14 (77.7)^a^	101 (50.5)
Acinar predominant	1 (5.6)	16 (8)
Invasive mucinous adenocarcinoma	1 (5.6)	4 (2)
Lepidic predominant	0 (0)	4 (2)
Solid predominant	0 (0)	15 (7.5)
Micropapillary predominant	2 (11.1)	0 (0)
Differentiation		
Well	10 (55.6)	170 (85)
Moderate	8 (44.4)^a^	11 (5.5)
Poor	0 (0)	19 (9.5)
Lymphatic invasion		
(+)	5 (27.8)	4 (2)
(−)	13 (72.2)^a^	196 (98)
Venous invasion		
(+)	0 (0)	4 (2)
(−)	18 (100)	196 (98)

a P<0.05 vs. the MPP-negative group. Data >3 are presented as the mean ± standard error of the mean.

AIS; adenocarcinoma *in situ*; MIA, minimally invasive adenocarcinoma; MPP, micropapillary pattern.

**Table II:** E-cadherin and vimentin expression in patients with adenocarcinoma in the apparent MPP component group and no MPP component group.

		Apparent MPP component group (n=18)		
			
Antibody	Grade	MPP component	Component without MPP	No MPP group (n=26)
E-cadherin	3+	18	18	21
	2+	0	0	5
	1+	0	0	0
	0	0	0	0
	3+	9	2	6
Vimentin	2+	7	3	3
	1+	2	1	4
	0	0	12^a^	13^a^

Grade of expression was defined according to the proportion of positive cells as follows: 0, p<5% positive cells; 1+, 5≤p<30% positive cells; 2+, 30≤p<70% positive cells; 3+, p≥70% positive cells.

a P<0.05 vs. MPP component in apparent MPP component group.

MPP, micropapillary pattern.

**Table III:** Vimentin expression of cancer cells invading in a lymph vessel.

		Expression of vimentin					
		
Case	p of MPP component area (%)	Component without MPP	MPP component	ly-1	ly-2	ly-3	ly-4
1	15≤p<30	2+	3+	3+	3+		
2	5≤p<15	1+	3+	2+			
3	5≤p<15	0	3+	2+	2+	2+	2+
4	15≤p<30	0	3+	2+			
5	5≤p<15	0	2+	2+	2+	2+	2+

p, proportion; ly, lymphatic vessel.

## References

[1] Dela Cruz CS, Tanoue LT, Matthay RA (2011). Lung cancer: epidemiology, etiology and prevention. Clin Chest Med.

[2] Amin MB, Tamboli P, Merchant SH (2002). Micropapillary component in lung adenocarcinoma: a distinctive histologic feature with possible prognostic significance. Am J Surg Pathol.

[3] Miyoshi T, Satoh Y, Okumura S (2003). Early-stage lung adenocarcinomas with a micropapillary pattern, a distinct pathologic marker for a significantly poor prognosis. Am J Surg Pathol.

[4] Kamiya K, Hayashi Y, Douguchi J (2008). Histopathological features and prognostic significance of the micropapillary pattern in lung adenocarcinoma. Mod Pathol.

[5] Postmus PE, Brambilla E, Chansky K (2007). The IASLC Lung Cancer Staging Project: proposals for revision of the M descriptors in the forthcoming (seventh) edition of the TNM classification of lung cancer. J Thorac Oncol.

[6] Travis WD, Brambilla E, Noguchi M (2011). International association for the study of lung cancer/american thoracic society/european respiratory society international multidisciplinary classification of lung adenocarcinoma. J Thorac Oncol.

[7] Kalluri R, Weinberg RA (2009). The basics of epithelial-mesenchymal transition. J Clin Invest.

[8] Liu T, Zhang X, Shang M (2013). Dysregulated expression of Slug, vimentin, and E-cadherin correlates with poor clinical outcome in patients with basal-like breast cancer. J Surg Oncol.

